# Feeling Pressure to Be a Perfect Mother Relates to Parental Burnout and Career Ambitions

**DOI:** 10.3389/fpsyg.2018.02113

**Published:** 2018-11-05

**Authors:** Loes Meeussen, Colette Van Laar

**Affiliations:** ^1^Center for Social and Cultural psychology, Psychology Department, KU Leuven, Leuven, Belgium; ^2^Fonds Wetenschappelijk Onderzoek, Brussels, Belgium

**Keywords:** perfect mothering norm, intensive mothering beliefs, parental burnout, stress, regulatory focus, maternal gatekeeping, work-family balance, career ambitions

## Abstract

**Background and aims:** Intensive mothering norms prescribe women to be perfect mothers. Recent research has shown that women’s experiences of pressure toward perfect parenting are related to higher levels of guilt and stress. The current paper follows up on this research with two aims: First, we examine how mothers regulate pressure toward perfect mothering affectively, cognitively, and behaviorally, and how such regulation may relate to parental burnout. Second, we examine how feeling pressure toward perfect mothering may spill over into mothers’ work outcomes.

**Methods:** Through Prolific Academic, an online survey was sent to fulltime working mothers in the United Kingdom and United States with at least one child living at home (*N* = 169). Data were analyzed using bootstrapping mediation models.

**Results:** Feeling pressure to be a perfect mother was positively related to parental burnout, and this relation was mediated by parental stress, by a stronger cognitive prevention focus aimed at avoiding mistakes as a mother, and by higher maternal gatekeeping behaviors taking over family tasks from one’s partner. Moreover, pressure toward perfect mothering had a positive direct effect on career ambitions; and a negative indirect effect, such that mothers with higher felt pressure toward perfect mothering experienced lower work-family balance, which in turn related to lower career ambitions.

**Conclusion:** The findings suggest that intensive mothering norms might have severe costs for women’s family and work outcomes, and provide insights into where to direct efforts to reduce motherhood hardships and protect women’s career ambitions.

## Introduction

The transition to parenthood is considered one of the major milestones in people’s lives that comes with great joy and happiness (Hansen, 2012). Yet, empirical evidence does not support such an increase in happiness for parents. While parenthood may benefit some outcomes such as life meaning, people with (especially younger) children often show lower life satisfaction and higher depressive symptoms than people without children (Evenson and Simon, 2005; Umberson et al., 2010; Hansen, 2012; Stanca, 2012). In fact, parenthood can even lead to exhaustion so much that parental burnout emerges (Roskam et al., 2017). Moreover, parents increasingly face the challenge to combine childcare tasks with a paid job as more and more women entered the labor market and there are more and more dual earning families (Cotter et al., 2008; Pew Research Center, 2015). This combination of work and family is not always easy, and parents have to make decisions regarding the extent to which they spend time and energy on their family or on pursuing career ambitions (Greenhaus and Beutell, 1985; Allen et al., 2000). In the current paper, we argue that one of the factors that may increase parental burnout in mothers and harm their career ambitions is the societal pressure to be a perfect mother.

### Pressure to Be a Perfect Mother

For women especially, Western societies prescribe motherhood as a central life goal through which one achieves womanhood (Christler, 2013), and a dominant discourse of ‘intensive mothering’ norms prescribes mothers to be the main one responsible to take care of the children and to be fully devoted to this task, putting the children’s needs before her own (Hays, 1996; Liss et al., 2013a; Newman and Henderson, 2014). Such social norms and expectations are highly influential for people’s affect, cognition, and behavior (Major, 1994; Cialdini and Trost, 1998). Being able to fulfill the norms of motherhood is important for the affirmation of this central social identity and one’s sense of self (Turner et al., 1987; Gaunt, 2008). Following social norms is also important for one’s need to belong (Baumeister and Leary, 1995), since people are socially rewarded when adhering to social norms, and punished when deviating from them. Indeed, research has shown that mothers fear social penalties when they fail to meet the high motherhood standards (Henderson et al., 2010; Liss et al., 2013b) and working mothers and mothers who decide not to take their full due maternity leave are evaluated by others as ‘bad parents’ and as less desirable partners (Okimoto and Heilman, 2012; Morgenroth and Heilman, 2017).

While such high expectations for mothers may stem from a positive view on motherhood, they can also have costs for today’s mothers. Research has shown that women’s feelings of being pressured to be a perfect mother are related to increased maternal guilt, lower self-efficacy beliefs, and higher stress levels - even when these women do not hold strong intensive mothering beliefs themselves (Rotkirch and Janhunen, 2009; Henderson et al., 2016; Borelli et al., 2017). The current research aims to increase our understanding of the effects of pressure to be a perfect mother on working mothers’ health, parental and work outcomes. First, we examine the different processes through which feeling pressure to be a perfect mother may lead to parental burnout. Going beyond commonly studied affective consequences of such pressure, we argue that feeling this pressure will also trigger cognitive and behavioral regulation strategies in mothers that over time may increase the risk of parental burnout. Second, we move beyond the family domain, arguing that feeling pressure to be a perfect mother can strain women’s work-family balance, which can in turn decrease their career ambitions.

### Pressure to Be a Perfect Mother and Parental Burnout

Parental burnout refers to the emotional exhaustion of parents, emotional distancing from their children, and reduced feelings of parental accomplishment and efficacy (Roskam et al., 2017). It has severe consequences for parents themselves, increasing escape and suicidal ideations, sleep problems, and addictions; as well as for their partner, increasing conflict and partner estrangement; and their children, increasing neglectful and violent behaviors toward the children (Mikolajczak et al., 2018a). So far, research on the antecedents of parental burnout has largely focused on the individual and the family level. At the individual level, parents high in neuroticism and attachment avoidance and parents low in conscientiousness, agreeableness, and emotional intelligence show higher risk of parental burnout (Le Vigouroux et al., 2017; Mikolajczak et al., 2018b). Also, parents low in positive parenting, self-efficacy beliefs, and parents who perceive their parental role to restrict their freedom are more at risk of parental burnout (Mikolajczak et al., 2018b). At the family level, family disfunction such as higher family disorganization and conflict, and lower co-parental agreement and marital satisfaction relate to a higher risk of parental burnout (Mikolajczak et al., 2018b). While it has been argued that parental burnout may also be affected by societal factors (Roskam et al., 2017; Mikolajczak et al., 2018b), so far these have not yet been studied. In the current study, we examine pressure to be a perfect mother as a societal-level antecedent of parental burnout.

### Mothers’ Cognitive and Behavioral Regulation of Pressure to Be a Perfect Mother

Previous research has shown that mothers can show negative affective reactions to the pressure to be a perfect mother such as increased stress or feelings of guilt (Rotkirch and Janhunen, 2009; Henderson et al., 2016; Borelli et al., 2017). Yet, mothers are not just passive recipients of intensive mothering norms, they likely also try to actively regulate such social pressure to be a perfect mother. To date, very little is known about how mothers regulate this pressure. Based on previous research on how people regulate overly high performance expectations in school and sports contexts (Stoll et al., 2008; Elison and Partridge, 2012), we argue that mothers will regulate their mother identity in similar ways.

Looking into cognitive regulation strategies, research has shown that overly high performance expectations induce a fear of failure and a concern over mistakes (Stoll et al., 2008; Elison and Partridge, 2012). In order to prevent mistakes and failure, people show a regulatory “prevention focus.” In a prevention focus, people pay increased attention to what can go wrong and how this can be prevented with the aim of avoiding undesired, negative outcomes (for a review, see Higgins, 1998). Such a cognitive strategy is commonly contrasted with a “promotion focus.” In a promotion focus, people pay increased attention to potential success and how to attain desired, positive outcomes (Higgins and Tykocinski, 1992). When mothers feel that their sense of self as a valued and successful mother is threatened - which is likely in face of overly high mothering standards - a prevention focus may be triggered to deal with such threat and to protect the mother identity and performance as a mother.

Looking into behavioral regulation strategies, research has shown that perfectionist standards are related to increased striving for excellent performance (Stoll et al., 2008). Within the mothering domain, women’s efforts for excellence may show through maternal gatekeeping behaviors as a way to affirm their mother identity (Gaunt, 2008). Maternal gatekeeping refers to women’s behavior of restricting their partner’s involvement in household and childcare by “guarding” the management of these tasks, doing tasks themselves, setting the standards of how tasks need to be done, and re-doing them to these standards after their partner performed a task (Allen and Hawkins, 1999; Puhlman and Pasley, 2013). Research has shown that mothers’ gatekeeping behaviors are stronger the more they themselves endorse intensive mothering beliefs (Liss et al., 2013a). Similarly, it is likely that not only women’s own high standards of mothering, but also socially prescribed standards of mothering (i.e., pressure to be a perfect mother) will trigger maternal gatekeeping behaviors in women - as they may feel they will be the ones judged for the quality of childcare in their family and aim to avoid social devaluation and protect their mother identity.

While both these regulation strategies may be functional reactions to pressure into being a perfect mother, and may affirm the mother identity and protect performance as a mother in the short run, we expect that these regulation strategies may also have costs over time. Research has shown that a prevention focus protects performance in the short run, but that it is highly depleting for people over time (Inzlicht et al., 2006; Schmader et al., 2008; Ståhl et al., 2012). Similarly, maternal gatekeeping behaviors may affirm one’s mother identity in the short run (Gaunt, 2008), but they also increase women’s portion of family tasks and hence their second shift (Hochschild and Machung, 2012). Therefore, we expect that not only women’s negative affective reactions to pressure to be a perfect mother, but also their cognitive and behavioral regulation of this pressure may make mothers more vulnerable to parental burnout.

### Pressure to Be a Perfect Mother and Career Ambitions

Research on intensive mothering and social pressure to be a perfect mother has mainly focused on how such high mothering standards influence women’s affective family outcomes and their general well-being. Yet, as women are increasingly combining work and family roles (Cotter et al., 2008), it is important to also investigate the extent to which the pressure to be a perfect mother relates to mothers’ work outcomes, which is the second aim of the current research. People’s work and family lives are closely linked, hence choices in one life domain can spill-over to the other domain (Bakker and Demerouti, 2013). Thus, increased efforts to fulfill one’s role as a mother are likely to involve costs to one’s work role (Eccles, 1994). Therefore, pressure to be a perfect mother may put a strain on women’s work-family balance, and as they make choices to be able to combine both life domains, women may decrease their career ambitions in order to fulfill the high expectations of mothering.

### Research Aims and Hypotheses

In sum, the current study aims to examine how pressure to be a perfect mother relates to working mothers’ parenting and work outcomes. First, we examine the different processes through which feeling pressure to be a perfect mother may relate to parental burnout. Going beyond commonly studied affective consequences of pressure to be a perfect mother, we argue that mothers also regulate such pressure cognitively and behaviorally, which in turn may relate to higher parental burnout. Second, we move beyond the family domain, arguing that feeling pressure to be a perfect mother is negatively related to women’s work-family balance, which in turn relates to lower career ambitions.

With these aims, the current study increases insight into the mechanisms through which gender inequalities in both work and family domains may be strengthened by intensive mothering discourses. In the family domain, pressure to be a perfect mother may reinforce women’s higher investment in childcare tasks as compared to men (European Institute for Gender Equality, 2015), since maternal gatekeeping behaviors increase women’s second shift (Hochschild and Machung, 2012) and restrict men’s opportunities to invest in domestic tasks (Mcbride et al., 2005; Gaunt, 2008). In the work domain, pressure to be a perfect mother may reinforce gender inequalities by contributing to women’s underrepresentation in positions of power, lower work participation rates, and lower pay (European Union, 2016).

Our two hypotheses are:

H1: Feeling pressure to be a perfect mother is positively related to parental stress, prevention focus (but not a promotion focus) as a mother, and maternal gatekeeping behaviors, which in turn all relate to higher parental burnout.

H2: Feeling pressure to be a perfect mother is negatively related to mothers’ career ambitions through reduced feelings of work-family balance.

In studying these relationships, we control for women’s own intensive mothering beliefs as we aim to disentangle the influence of experienced social pressure to be a perfect mother from women’s own perfectionism in being a mother. This is an important distinction, as previous research has shown that women’s own intensive motherhood beliefs are related to lower life satisfaction and higher depression and stress (Rizzo et al., 2013) and that own perfectionism is related to parental stress, burnout, and work-family conflict (Mitchelson and Burns, 1998; Mitchelson, 2009; Hill and Curran, 2016). Speaking to the importance of social pressure above one’s own standards, research has shown that pressure to be a perfect mother has affective consequences controlling for effects of women’s own intensive mothering beliefs (Henderson et al., 2016), and research on perfectionism shows that perfectionism focused on societal expectations has more negative consequences for well-being (e.g., increased stress and burnout) than perfectionism focused on one’s own expectations (Mitchelson and Burns, 1998; Lee et al., 2012; Hill and Curran, 2016; Kawamoto and Furutani, 2018).

## Materials and Methods

### Procedure

We investigated the relation between pressure to be a perfect mother and parental burnout and career ambitions using an online survey presented as a study “interested in people’s daily lives and how they feel about family and work.” Data was collected in October-November 2017 through Prolific Academic, an online participant recruitment website supported by Oxford University^1^ which, in comparison to other online research platforms, generally provides good quality data in terms of scale reliability, replication of known effects, passing attention checks, participants’ (low) familiarity with measures, and (low) dishonesty of participants (Peer et al., 2017). Prolific Academic recruits participants primarily via social media (Facebook, Twitter, Reddit, blog posts), via poster and flyer campaigns at universities, and through word of mouth. Thus, participants self-select to be part of this online participant recruitment website. When posting a study on this website, Prolific sends an email to a random subset of all eligible participants (based on selection criteria indicated by the researchers). Participants also receive a weekly newsletter with a list of studies in which they are eligible to participate in. Of the Prolific participant pool (*N* = 38579 at the time of data recruitment^2^), we used the following selection criteria: full-time working, female participants currently living in the United Kingdom or United States, who have at least one child, with the first (or only) child born between 1996 and 2017 (to aim for a large enough participant pool with at least one child living at home). This resulted in 1477 eligible participants. The study was approved by the Social and Societal Ethics Committee of the University of Leuven, Belgium (registration number G-201709929). The first page of the online questionnaire consisted of the informed consent form with a study description, information about confidentiality, voluntary participation, the possibility to quit at any time, and contact information of the researchers and ethics committee. Only when participants indicated they had read this information and agreed to participate could they continue to the questionnaire. Participants received 1.25 British pounds upon completion of the survey, which took approximately 15 min. Initially, we intended to manipulate pressure to be a perfect mother using a fictitious newspaper article (intensive mothering norm, more attainable mothering norm, or control condition without article). Yet, this manipulation failed to affect mothers’ feelings of pressure toward perfect mothering or family and work outcomes, suggesting that the manipulation was not strong enough to overrule the daily normative pressures mothers experience in their social environment. Further information about this manipulation and analyses of its (null) effects can be obtained from the first author upon request. All analyses presented in this paper fully replicate when controlling for condition, which did not have significant relations with any of the variables.

### Participants

Of the 186 participants, ten were removed because they failed to answer one or both of the attention checks correctly (e.g., ‘When you read this item, please select the point on the scale that indicates “two”) and seven were removed because they indicated their children no longer lived at home. The remaining 169 working mothers^3^ were 36.74 years old on average (*SD* = 7.62, range 19.00–58.00). The majority (88.2%) lived in the United Kingdom and 11.8% in the United States. Participants had one to five children (*M* = 1.82, *SD* = 0.88) with the age of the youngest child ranging from zero to 20 (*M* = 6.68, *SD* = 5.49). 84.6% of all participants were currently in a relationship, 64.3% of whom were married. Education levels extended across the full range, with 21.3% having obtained a high school degree, 26.6% a college or associate’s degree, 36.7% a bachelor’s degree, 13.6% a master’s degree, and 1.8% a Ph.D. degree. On average, participants were active on the labor market for 14.69 years (*SD* = 7.61, range 1.00–40.00) and worked 39.15 h per week (*SD* = 7.20, range 30.00–80.00).

### Measures

All items were answered on a 7-point scale from (1) strongly disagree to (7) strongly agree unless otherwise indicated. Measures were scored such that higher scores indicate more of the concept.

#### Pressure to Be a Perfect Mother

Participants indicated feelings of pressure to be a perfect mother by rating to what extent they agreed with the statements: “I feel pressured to be ‘perfect’ in my role as a mother” and “My social environment sets very high expectations for me as a mother to live up to” (items based on Henderson et al., 2010, 2016 who studied similar samples)^4^.

#### Parental Stress

Participants were asked to think about themselves as a mother and to indicate to what extent they feel anxious, distressed, worried, and tense (1-not at all to 7-very much) (based on the validated PANAS scale by Watson et al., 1988, and used in a similar sample and in the context of family and work life by Van Steenbergen et al., 2008).

#### Prevention Focus as a Mother

Mothers’ prevention focus was measured with three items from the Regulatory Focus Scale (Lockwood et al., 2002) adapted to apply to motherhood: “I am anxious that I will fall short of my responsibilities and obligations as a mother,” “I think about how I can prevent failures as a mother,” and “I focus on avoiding mistakes as a mother.”

#### Promotion Focus as a Mother

Mothers’ promotion focus was measured with three items from the Regulatory Focus Scale (Lockwood et al., 2002) adapted to apply to motherhood: “I think about how I can realize my hopes, wishes, and aspirations as a mother,” “I focus on achieving positive outcomes as a mother,” and “I think about how I can be a really good mother.”

#### Maternal Gatekeeping

Maternal gatekeeping behaviors were measured with three items from the Maternal Gatekeeping measure by Allen and Hawkins (1999), (validated in similar sample) applied to childcare tasks: “I have higher standards than my partner for how well cared for the child(ren) should be,” “I like to be in charge when it comes to childcare responsibilities,” and “It’s hard to teach my partner the skills necessary to do certain childcare tasks right, so I’d rather do them myself.”

#### Parental Burnout

We used four items from the Parental Burnout Inventory (Roskam et al., 2017) to measure parental burnout (scale validated in a similar but mixed-gender sample): “I feel emotionally drained by my mother role,” “I sometimes feel as though I am taking care of my child(ren) on autopilot,” “I feel tired when I get up in the morning and have to face another day with my children,” and “I am at the end of my patience at the end of a day with my child(ren)”^5^.

#### Work-Family Balance

The extent to which participants felt that their work and family domains were in balance was measured with three items (based on Hill et al., 2001, who use this scale among working men and women, 63% of whom had children): “It is easy for me to balance the demands of my work and my family life,” “All in all, I am successful in balancing my family life and my work,” and “I am able to find a good balance between work and family.”

#### Career Ambitions

Career ambitions were assessed with five items asking participants to what extent they would like to pursue certain work aspirations in the near future: “In the near future, I would like to… perform better at work, take up more tasks at work, pursue a higher position at work, pursue a promotion, increase my responsibilities at work.”

#### Own Intensive Mothering Beliefs

Own intensive mothering beliefs (control variable) were measured with eight items from the Intensive Parenting Attitudes Measure (Liss et al., 2013a, validated in a similar sample) covering the dimensions of essentialism (e.g., “Although fathers may mean well, they generally are not as good at parenting as mothers”), fulfillment (e.g., “Being a parent brings a person the greatest joy he or she can possibly experience”), stimulation (e.g., “It is important for parents to interact regularly with children on their level, e.g., getting down on the floor and playing with them”), and child-centeredness (e.g., “Children’s needs should come before their parents”) with two items each.

Table 1 provides reliability scores, means, standard deviations, and correlations between all measures.

**Table 1:** Descriptives of and correlations between all measures.

		α	*M* (*SD*)	1	2	3	4	5	6	7	8
1. Pressure to be a perfect mother		0.75	4.00 (1.70)								
2. Parental stress		0.84	3.42 (1.33)	0.49***							
3. Prevention focus as a mother		0.63	4.49 (1.26)	0.42***	0.39***						
4. Promotion focus as a mother		0.84	5.40 (1.13)	0.15	0.05	0.43***					
5. Maternal gatekeeping		0.71	4.39 (1.58)	0.34***	0.26**	0.16*	0.08				
6. Parental burnout		0.79	3.34 (1.44)	0.34***	0.40***	0.21**	-0.31***	0.28***			
7. Work-family balance		0.93	4.61 (1.49)	-0.20*	-0.31***	-0.25**	0.15	-0.12	-0.25**		
8. Career ambitions		0.88	4.35 (1.53)	0.13	0.08	0.02	0.13	-0.05	-0.04	0.26**	
9. Own intensive mothering beliefs		0.76	4.92 (0.98)	0.28***	0.13	0.20**	0.29***	0.40***	-0.03	0.11	0.13


*^∗∗∗^*p* < 0.001, ^∗∗^*p* < 0.01, and ^∗^*p* < 0.05.*

### Analyses

We analyzed the data with two bootstrapping mediation models (5000 samples) using the PROCESS macro in SPSS (Hayes, 2013). Model 1 tested Hypothesis 1, with pressure to be a perfect mother as the independent variable, parental burnout as the dependent variable, and parental stress, prevention and promotion focus, and maternal gatekeeping as mediators, while controlling for own intensive mothering beliefs. Model 2 tested Hypothesis 2, with pressure to be a perfect mother as the independent variable, career ambitions as the dependent variable, and work-family balance as the mediator, while controlling for own intensive mothering beliefs. We also controlled for number of children, age of the youngest child, relationship status, education, work hours per week, and country.

## Results

### Hypothesis 1: Pressure to Be a Perfect Mother Is Positively Related to Parental Burnout, and This Relation Is Mediated by Parental Stress, Prevention Focus as a Mother, and Maternal Gatekeeping Behaviors

**FIGURE 1:**
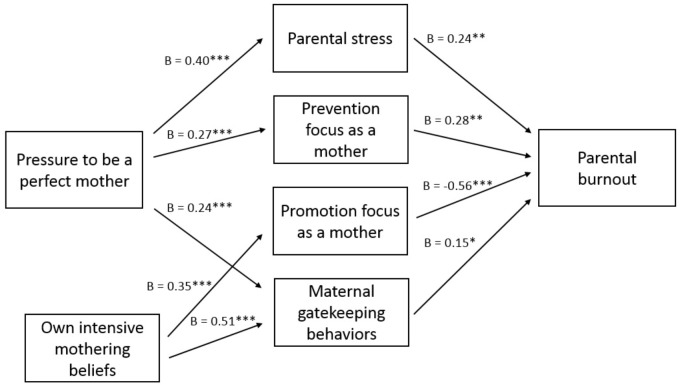
Graphical overview of significant paths in Model 1: Parental stress, prevention focus, and maternal gatekeeping as mediators between pressure to be a perfect mother and parental burnout (^∗∗∗^*p* < 0.001, ^∗∗^*p* < 0.01, and ^∗^*p* < 0.05).

First, we hypothesized that feeling pressure to be a perfect mother would be related to higher levels of parental burnout, and that this relationship would be mediated by parental stress, prevention focus (but not a promotion focus) as a mother and maternal gatekeeping behaviors. Table 2 provides an overview of all tested relations in Model 1, and Figure 1 gives a graphical overview of the significant paths (without the control variables number of children, age of the youngest child, relationship status, education, work hours per week, and country for the sake of clarity). Results showed that feeling pressure to be a perfect mother was indeed positively related to parental burnout (β = 0.14, *p* = 0.029). Moreover, this relationship was significantly mediated by higher levels of parental stress [indirect effect: 0.10, 95% CI (0.04, 0.17)], prevention focus as a mother [indirect effect: 0.08, 95% CI (0.03, 0.14)], and maternal gatekeeping behaviors [indirect effect: 0.04, 95% CI (0.01, 0.09)]. While a promotion focus was negatively related to parental burnout (β = -0.56, *p* < 0.001), it was not related to feeling pressure to be a perfect mother. Thus, mothers who experienced more pressure to be a perfect mother experienced more parental stress, had a stronger prevention focus trying to avoid mistakes as a mother, and showed more maternal gatekeeping behaviors taking over childcare tasks from their partner, and these in turn all related to higher levels of parental burnout.

**Table 2:** Results of Model 1: Parental stress, prevention focus, and maternal gatekeeping as mediators between pressure to be a perfect mother and parental burnout.

			Parental stress			Prevention focus			Promotion focus			Maternal gatekeeping			Parental burnout	
											
Direct paths			*B(SE)*	*p*		*B(SE)*	*p*		*B(SE)*	*p*		*B(SE)*	*p*		*B(SE)*	*p*
Variables of interest																
	Pressure to be a perfect mother		0.40 (0.06)	0.000		0.27 (0.06)	0.000		0.01 (0.05)	0.807		0.24 (0.07)	0.000		0.14 (0.07)	0.029
	Parental stress														0.24 (0.08)	0.003
	Prevention focus as a mother														0.28 (0.09)	0.002
	Promotion focus as a mother														-0.56 (0.10)	0.000
	Maternal gatekeeping														0.15 (0.06)	0.021
																
Control variables																
	Own intensive mothering beliefs		-0.02 (0.10)	0.815		0.13 (0.10)	0.169		0.35 (0.09)	0.000		0.51 (0.12)	0.000		-0.16 (0.11)	0.132
	Number of children		0.05 (0.10)	0.649		0.004 (0.10)	0.968		-0.12 (0.09)	0.192		0.23 (0.12)	0.063		0.06 (0.10)	0.536
	Age of the youngest child		0.001 (0.02)	0.958		-0.001 (0.02)	0.945		0.000 (0.02)	0.985		-0.03 (0.02)	0.128		-0.05 (0.02)	0.002
	Relationship status (1 = in relation)		-0.21 (0.26)	0.423		0.41 (0.26)	0.120		0.44 (0.23)	0.057		-0.67 (0.31)	0.032		-0.18 (0.26)	0.481
	Education		-0.08 (0.09)	0.374		0.05 (0.09)	0.580		0.17 (0.08)	0.035		0.09 (0.11)	0.426		-0.09 (0.09)	0.290
	Work hours per week		0.01 (0.01)	0.408		0.01 (0.01)	0.253		-0.01 (0.01)	0.255		-0.01 (0.02)	0.605		-0.03 (0.01)	0.047
	Country (1 = United Kingdom)		0.44 (0.28)	0.124		0.09 (0.28)	0.748		-0.92 (0.25)	0.000		0.30 (0.33)	0.369		-0.17 (0.29)	0.557
																
	*R*^2^		0.26, *p* < 0.001			0.21, *p* < 0.001			0.21, *p* < 0.001			0.27, *p* < 0.001			0.44, *p* < 0.001	
																

**Indirect paths**			**Estimate (*SE*)**			**95% CI**										

	Parental stress		0.10 (0.03)			[0.04, 0.17]										
	Prevention focus as a mother		0.08 (0.03)			[0.03, 0.14]										
	Promotion focus as a mother		-0.01 (0.03)			[-0.07, 0.05]										
	Maternal gatekeeping		0.04 (0.02)			[0.01, 0.09]										


These results were found while controlling for participants’ own intensive mothering beliefs, number of children, age of the youngest child, relationship status, education, work hours per week, and country. These control variables showed no significant relations with the four mediators nor with parental burnout, with a few exceptions: participants who had higher intensive mothering beliefs, participants from the United States, and participants with higher education levels had a higher promotion focus as a mother (β = 0.35, *p* < 0.001; β = -0.92, *p* < 0.001; and β = 0.17, *p* = 0.035, respectively), participants who were not in a relationship and participants who had higher intensive mothering beliefs showed more maternal gatekeeping behaviors (β = -0.67, *p* = 0.032 and β = 0.51, *p* < 0.001, respectively), and participants who worked more hours per week and participants with older children reported lower levels of parental burnout (β = -0.03, *p* = 0.047 and β = -0.05, *p* = 0.002, respectively).

### Hypothesis 2: Pressure to Be a Perfect Mother Is Negatively Related to Career Ambitions, and This Relation Is Mediated by Work-Family Balance Experiences

**FIGURE 2:**
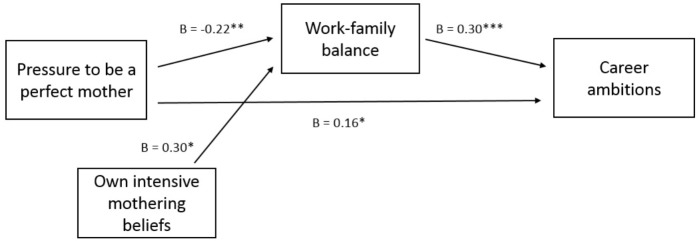
Graphical overview of significant paths in Model 2: Work-family balance as a mediator between pressure to be a perfect mother and career ambitions (^∗∗∗^*p* < 0.001, ^∗∗^*p* < 0.01 and ^∗^*p* < 0.05).

Second, we hypothesized that feeling pressure to be a perfect mother would be negatively related to mothers’ career ambitions through reduced feelings of work-family balance. Table 3 provides an overview of all tested relations in Model 2, and Figure 2 gives a graphical overview of the significant paths (without the control variables number of children, age of the youngest child, relationship status, education, work hours per week, and country for the sake of clarity). Results showed an unexpected positive direct relation between pressure to be a perfect mother and career ambitions (β = 0.16, *p* = 0.036; on which we elaborate in the discussion section); as well as the hypothesized negative indirect relation between pressure to be a perfect mother and career ambitions through work-family balance [indirect effect: -0.06, 95% CI (-0.15, -0.01)]: mothers who felt more pressure to be a perfect mother experienced a lower balance between their work and family domains (β = -0.22, *p* = 0.002), and in turn had lower career ambitions (β = 0.30, *p* < 0.001).

**Table 3:** Results of Model 2: Work-family balance as a mediator between pressure to be a perfect mother and career ambitions.

			Work-family balance		Career ambitions	
				
Direct paths			*B*(SE)	*p*	*B*(SE)	*p*
Variables of interest						
	Pressure to be a perfect mother		-0.22 (0.07)	0.002	0.16 (0.07)	0.036
	Work-family balance				0.30 (0.08)	0.000
Control variables						
	Own intensive mothering beliefs		0.30 (0.12)	0.013	0.11 (0.13)	0.397
	Number of children		0.04 (0.13)	0.738	-0.13 (0.13)	0.315
	Age of the youngest child		0.04 (0.02)	0.090	0.01 (0.02)	0.641
	Relationship status (1 = in relation)		0.13 (0.32)	0.691	0.28 (0.33)	0.401
	Education		0.12 (0.11)	0.301	0.03 (0.11)	0.782
	Work hours per week		-0.02 (0.02)	0.225	-0.01 (0.02)	0.604
	Country (1 = United Kingdom)		-0.86 (0.35)	0.014	0.08 (0.36)	0.832
						
	*R*^2^		0.13, *p* = 0.005		0.12, *p* = 0.016	
						

**Indirect path**			**Estimate (*SE*)**		**95% CI**	

	Work-family balance		-0.06 (0.03)		[-0.15, -0.01]	


This model also controlled for mothers’ own intensive mothering beliefs, number of children, age of the youngest child, relationship status, education, work hours per week, and country, which were unrelated to work-family balance experiences and career ambitions, with two exceptions: Participants with higher intensive mothering beliefs and participants in the United States had more positive work-family balance experiences (β = 0.30, *p* = 0.013 and β = -0.86, *p* = 0.014, respectively).

## Discussion

The current paper investigated how intensive mothering norms relate to mothers’ parental and work outcomes. First, we investigated mothers’ affective, cognitive, and behavioral regulation in the face of pressure to be a perfect mother, and how these in turn related to parental burnout. Second, we investigated how pressure to be a perfect mother related to mothers’ career ambitions via their work-family balance experiences.

### Regulating Pressure to Be a Perfect Mother and Parental Burnout

The findings show that feeling pressure to be a perfect mother relates to affective reactions in mothers with increased parental stress (in line with Borelli et al., 2017; Henderson et al., 2016; Rizzo et al., 2013), but also to cognitive regulation (higher prevention focus – aiming to avoid mistakes as a mother) and behavioral regulation (maternal gatekeeping behaviors - taking over childcare tasks from their partner). While this regulation could be functional in adhering to the high standards of motherhood and affirming one’s mother identity (Gaunt, 2008), this regulation may also have costs: the results showed that these affective, cognitive, and behavioral reactions all related to higher parental burnout. These findings contribute to previous research on parental burnout by indicating that parental burnout may not only be triggered by individual and family-level risk factors (Le Vigouroux et al., 2017; Mikolajczak et al., 2018b), but potentially also by intensive mothering norms at the societal level.

These findings also suggest that while normative pressure for women to be perfect mothers may have the purpose of increasing maternal investment and improving children’s well-being and health (Cicchetti and Toth, 1998; Repetti et al., 2002; Sutherland, 2010), this pressure could risk the opposite effect: research has shown that children’s development is harmed when their mother suffers from mental health problems (Beardslee et al., 1983; Cummings and Davies, 1994), parental burnout is related to neglectful and violent behavior toward one’s children (Mikolajczak et al., 2018a), and children experience more depressive symptoms and lower life satisfaction the more their parents experienced pressure to be perfect as a parent (Randall et al., 2015).

### Pressure to Be a Perfect Mother and Career Ambitions

Additionally, the current study extended previous research by showing that feeling pressure to be a perfect mother not only relates to mothers’ family outcomes but also to their work outcomes. Specifically, we found that feeling such pressure was related to lower work-family balance experiences, which in turn were related to lower career ambitions in mothers. While the indirect effect of pressure to be a perfect mother on career ambitions through work family balance was indeed negative, there was also an unexpected positive direct relation between pressure to be a perfect mother and career ambitions. We interpret this as an indication that women with higher career ambitions may experience more social pressure to be a perfect mother, because social norms prescribe women to prioritize family over work (Meeussen et al., 2016; Haines et al., 2017) and diverging from such norms triggers social mechanisms (e.g., backlash, negative evaluations) as a pressure to conform (Rudman and Glick, 2001; Rudman et al., 2012; Morgenroth and Heilman, 2017). This positive direct relation between pressure to be a perfect mother and career ambitions is also in line with research showing a positive relation between the extent to which women value work success and the importance they attribute to motherhood (McQuillan et al., 2008). Mothers may thus compensate career ambitions with increased investment in their role as a parent. The combination of this positive direct and negative indirect relation then suggests that the mechanism outlined in our results (i.e., social pressure to be a perfect mother relates to lower work-family balance experiences, which in turn relate to lower career ambitions) may be triggered more for women with higher career ambitions. As such, there may be a buffering loop between career ambitions and pressure to be a perfect mother (women with higher career ambitions experience more pressure to be a perfect mother, which decreases their ambitions by putting a strain on their work-family balance) to avoid gender role incongruences. In future research, it would be interesting to examine whether women with higher career ambitions indeed are more pressured or feel more pressured to be a perfect mother.

### Implications

The current findings speak to the mechanisms through which gender inequalities in both work and family domains may be strengthened by intensive mothering discourses. Our findings showed that experienced pressure to be a perfect mother is related to higher maternal gatekeeping behaviors in mothers. As such, women share in childcare tasks is increased (Hochschild and Machung, 2012; European Institute for Gender Equality, 2015) and men’s opportunities to invest in domestic tasks are limited (Mcbride et al., 2005; Gaunt, 2008). For women, maternal gatekeeping behaviors may be functional to live up to intensive mothering expectations, but higher parental stress and focus on avoiding mistakes suggest that such increased investment in their family may not always be a positive experience, and even relates to parental burnout. For fathers, reduced opportunities to engage in childcare activities deprives them of the benefits of commitment and involvement with one’s children on fathers’ well-being (Knoester et al., 2007; see also Croft et al., 2015 for a review). For children, lower father involvement affects social and cognitive development (Marsiglio et al., 2000), and research has shown that families in which domestic labor is distributed unevenly among parents are less cohesive (Stevens et al., 2001).

Additionally, pressure to be a perfect mother may reinforce gender inequalities in the work domain. We find that women experience a lower work-family balance the more they feel pressure to be a perfect mother, and that this lower experienced balance is related to lower career ambitions. While this too can be a functional response allowing women to live up to high mothering expectations, it also has its costs: women’s underrepresentation in positions of power, lower work participation rates, and lower pay gives them less influence in decision-making in organizations and in politics, and makes women less economically independent than men, putting them at greater risk of poverty (European Union, 2016). At the societal level, female talents may be lost through these processes, limiting countries’ economic growth (Organization for Economic Cooperation and Development [OECD], 2012).

Importantly, the current study showed effects of social pressure to be a perfect mother while controlling for mothers’ own intensive mothering beliefs. Thus, even when mothers may appear to *choose* to take over childcare tasks from their partner or reduce their career ambitions, it is important to stress that such choices are (also) driven by social norms in their environment. This corroborates recent calls to avoid the strong rhetoric of “choice” in women’s home versus career decision making, as such a rhetoric fails to recognize the structural barriers women face and thus need to regulate – and even risks strengthening the belief that structural gender inequalities are no longer a problem (Stephens and Levine, 2011; van Engen et al., 2012; Vinkenburg, 2015).

## Limitations and Future Research

A limitation of the current study is that the data are correlational, so it is not possible to draw final causal conclusions. While it is likely that the pressure to be a perfect mother leads to parental burnout through parental stress, prevention focus as a mother, and maternal gatekeeping, the reverse may also occur: mothers with higher parental burnout may also feel more parental stress, show an increased prevention focus as a mother, try to instill a sense of control through maternal gatekeeping behaviors, and feel more pressure from their social environment. Similarly, as discussed above, the positive direct relation between feeling pressure to be a perfect mother and career ambitions suggests a reversed causal path between these variables such that women with higher career ambitions may experience higher pressure to be a perfect mother. Yet it is less likely that the indirect effect could be reversed such that career ambitions increase work-family balance experiences. We would expect that both causal directions are occurring to some extent and may create reinforcing or buffering feedback loops over time. Our attempt to experimentally manipulate pressure to be a perfect mother using fictitious articles was not successful, suggesting such a manipulation was not strong enough to overrule the daily normative pressures mothers experience in their social environment. Thus, future research could look into causal processes by either seeking stronger experimental manipulations or by using longitudinal studies, allowing more confident causal interpretations.

Our study design also has some limitations. First, participants were selected through the online participant recruitment website Prolific Academic. Research has shown that Prolific Academic generally provides good quality data (Peer et al., 2017), and our participants extended across a broad range in age, number and age of children, education level, job tenure, and work hours per week. Still, this is a sample of people who self-selected into being part of an online recruitment website and in participating in a study on “your daily life.” Results may thus not be generalizable to all full-time working mothers in the United Kingdom and United States. We recommend future research to look into the influence of pressure to be a perfect mother in more representative samples. Second, the measures of pressure to be a perfect mother, work-family balance, and career ambitions used in this study were not tested for validity and reliability in previous research. While internal consistency of these measures was good in our sample (α’s of 0.75, 0.93, and 0.88, respectively), future research should test the reliability of our findings with other validated scales. We especially encourage the development of a full scale measuring pressure to be a perfect mother tested for validity and reliability, which, to our knowledge, does not yet exist.

Another interesting route for future research is to investigate how mothers could be protected from parental burnout and decreased career ambitions. The current data show that mothers with a promotion focus –who focus on success and attaining desired, positive outcomes as a mother – show significantly lower parental burnout. Thus, there may be potential in trying to increase a promotion focus in mothers. While participants with stronger intensive mothering beliefs had a higher promotion focus, strengthening intensive mothering beliefs in mothers in order to instill a promotion focus may not be the best strategy since previous research has shown that mothers’ own intensive mothering beliefs are related to lower life satisfaction and higher depression and stress (Rizzo et al., 2013). Women may also differ in the extent to which they find it important to follow social expectations or care about social devaluation when they deviate from these expectations. Future research could thus investigate such individual differences as buffers that may mitigate the relationship between feeling pressure to be a perfect mother and parental and work outcomes, and potentially test interventions that support mothers in handling or resisting social pressure and to follow their own mothering beliefs and standards.

In addition to investigating factors that may help women cope with pressure to be a perfect mother, it could also be investigated how social pressure to be a perfect mother can be reduced. Research has shown that such pressure may come from women’s proximal social context. For instance, fellow mothers have been shown to be important sources of pressure to be a perfect mother (Henderson et al., 2010). Increased insight into what drives mothers to put such pressure on each other could point to possible ways to decrease this ‘interpersonal surveillance’. It is likely that a societal discourse of bias and backlash against mothers who do not fulfill intensive mothering ideals (Okimoto and Heilman, 2012; Morgenroth and Heilman, 2017) is driving such a ‘tug of war’ amongst mothers as mothers try to justify their own mothering behaviors and choices to protect their identity as a ‘good mother’ (Johnston and Swanson, 2006; Williams et al., 2016). Therefore, it may be more fruitful to tackle intensive mothering discourses at a structural level. The fact that participants in the current study (who were mainly from the United Kingdom) showed lower levels of pressure to be a perfect mother than participants from the United States in the studies by Henderson et al. (2010, 2016) also suggests that societal factors may be important in increasing or decreasing pressure on mothers. For instance, mass media can be an important source of cultural pressure to be a perfect mother, and this is a factor that could be altered (Henderson et al., 2010). A nice example is the action “#temporarilyunavailable” (#ikbenerevenniet in Dutch) by WomenInc, a Dutch organization that strives for gender equality. With this hashtag, more than 45 000 women responded to the call to plan a moment of me-time away from care tasks. Also, governmental policies can play an important role: when mothers receive much more parental leave than fathers after the birth of a child, this signals a social norm of mothers as those primarily responsible for taking care of the children. Similarly, as fathers receive less time to take up childcare activities, gender differences in actual childcare abilities are likely to be initiated or reinforced. Moreover, since intensive mothering norms have mainly been studied in Western societies, it would be interesting to investigate the extent to which mothers experience pressure to be a perfect mother in other regions, and whether such pressure triggers similar or different reactions in mothers across different regions. We are currently looking into the role of different national policies on mothers’ and fathers’ experienced pressure to be a perfect parent as well as their involvement in childcare in over 50 countries across the world.

Of course, proximal and more distal societal environments can not only reinforce intensive mothering discourses, but can also provide effective practical and psychological support for mothers for dealing with feeling pressure to be a perfect mother. At the proximal level, such support could be grandparents helping with childcare - signaling that the mother does not need to be the sole caregiver; or friends sharing their struggles and mistakes as a mother -signaling that it is ok to not always be able to live up to intensive mothering standards. A recent content analysis of online motherhood platforms shows that mothers do share negative feelings and matters concerning their own well-being, but preferably in closed groups, while positive feelings tend to be shared on public pages, perpetuating the intensive motherhood norm (César et al., 2018). At a more structural level, such support can come in the form of high quality childcare that allows mothers to outsource childcare tasks without feeling that this reduces the quality of care for their children; or programs that teach couples strategies to counter social pressures in parenting and to divide domestic tasks more equally in their relationship (see also Cowdery and Knudson-Martin, 2005). Also, organizations, trade-unions, and governments could aim to strengthen policies that facilitate the combination of work and family roles (for both mothers and fathers) to protect parents’ career ambitions, for example by offering work-family flexibility options such as working from home or by adapting meeting hours to school hours. Future research can thus investigate the extent to which such supports can buffer negative effects of feeling pressure to be a perfect mother, thus also providing insight into what forms of support can best be provided and strengthened.

Lastly, the current research can be extended to men and to the work domain. As both men and women are increasingly combining work and family roles, men too may be increasingly exposed to higher standards to be a father (Meeussen et al., 2016; Williams et al., 2016). Moreover, both men and women may also experience pressure from their work environment to be perfect in their job, a discourse called “the ideal worker” or “the work devotion scheme” (Williams et al., 2013). The combination of these two pressures puts working parents at risk for both parental burnout and work burnout. Future research could investigate the extent to which these two pressures co-occur for men and women, increasing a norm to ‘have it all’ (Hoffnung, 2004), and how these pressures then affect people’s work and family choices, experiences, and outcomes.

## Conclusion

In sum, the current research showed that for full-time working mothers who chose to participate in an online survey on Prolific Academic, experienced pressure to be a perfect mother was related to higher parental burnout in mothers through parental stress, prevention focus as a mother, and maternal gatekeeping behaviors. Moreover, pressure to be a perfect mother had a positive direct relation to career ambitions and a negative indirect relation through lower work-family balance experiences. These findings provide insights into where to direct efforts to reduce motherhood hardships and protect women’s career ambitions.

## Ethics Statement

This study was carried out in accordance with the recommendations of the University of Leuven Social and Societal Ethics Committee with written informed consent from all subjects. All subjects gave written informed consent in accordance with the Declaration of Helsinki. The protocol was approved by the University of Leuven Social and Societal Ethics Committee.

## Author Contributions

LM and CVL developed the research questions and designed the study. LM collected data, performed the analyses, and drafted the manuscript. LM and CVL worked on manuscript revisions and approved the final version of the manuscript for submission.

## Conflict of Interest Statement

The authors declare that the research was conducted in the absence of any commercial or financial relationships that could be construed as a potential conflict of interest.

## Footnotes

1 www.prolific.ac

2 https://app.prolific.ac/demographics for a full description

3 As indicated in the methods section, we initially intended to manipulate pressure to be a perfect mother using a fictitious newspaper article (intensive mothering norm, more attainable mothering norm, or control condition without article). The sample size was determined using an *a priori* power analyses, which estimated that we needed about 64 participants per condition to obtain statistical power at the recommended 0.80 level (Cohen, 1988) based on previously found medium effect sizes (Henderson et al., 2016). Since the manipulations failed to overrule the daily normative pressures mothers experience and we use the correlational data in the current paper, we performed a *post hoc* power analysis using G^∗^Power (Faul et al., 2007), which indicated that our sample size is sufficient to capture the current effect sizes [ΔR2 for models with and without mediator(s) is 0.224 for hypothesis 1 and 0.056 for hypothesis 2] with power of 100% for hypothesis 1 and 88.24% for hypothesis 2.

4 We did not include the item ‘I feel guilty when I cannot live up to the expectations for my parenting that I set for myself.’ as used by Henderson et al. (2010, 2016), because this item reflects own intensive motherhood standards rather than those in one’s social environment.

5 Item selection was based on the criteria of high factor loadings in the original scale (Roskam et al., 2017) on the one hand; and the potential to be affected by a manipulation of intensive mothering on the other hand (cf. attempted manipulation of pressure to be a perfect mother as described in the procedure section).
